# RIP3 deficiency ameliorates inflammatory response in mice infected with influenza H7N9 virus infection

**DOI:** 10.18632/oncotarget.16016

**Published:** 2017-03-08

**Authors:** Yu-Lin Xu, Hai-Lin Tang, Hao-Ran Peng, Ping Zhao, Zhong-Tian Qi, Wen Wang

**Affiliations:** ^1^ Department of Microbiology, Shanghai Key Laboratory of Medical Biodefense, Second Military Medical University, Shanghai, China

**Keywords:** influenza A virus, H7N9 virus, RIP3, necroptosis, proinflammatory cytokines, Immunology and Microbiology Section, Immune response, Immunity

## Abstract

Influenza H7N9 virus infection causes an acute, highly contagious respiratory illness that triggers cell death of infected cells and airway epithelial destruction. RIP3 is a key regulator of cell death responses to a growing number of viral and microbial agents. This study aimed to investigate the role of RIP3 in inflammation of influenza H7N9 virus infection. Here, RIP3 knock out (RIP3−/−) mice and littermate wild type mice were infected intranasally with influenza H7N9 virus (A/Fujian/S03/2015) to determine the contribution of RIP3 to the inflammatory response of influenza H7N9 virus infection. It was found that RIP3−/− mice infected with H7N9 virus showed higher survival and less weight loss, compared with wild type littermate mice. In addition, RIP3−/− mice had fewer regions of edema, infiltration with inflammatory cells, and alveolar collapses, and the secretions of IL-1β, IL-6, RANTES and MIP-1 in BALF were significantly decreased on days 3 and 7 p.i. when compared with WT mice. Moreover, caspase 1/IL1β signaling was found to be involved in RIP3 associated inflammation of influenza H7N9 virus, but not RIP3/MLKL dependent necrosis. In the conclusion, our results indicated that RIP3 deficiency can protect mice from the infection of influenza H7N9 virus by downregulating caspase 1/IL1β signaling, which provided evidence of the RIP3 involved necroptosis independent manner.

## INTRODUCTION

Human infection with avian influenza H7N9 virus was firstly reported in Feb, 2013 in China [1]. The emerging influenza virus had caused sporadic and reappeared infections with 440 laboratory-confirmed cases of which 122 deaths as of May, 2014, and remained a threat to human health and a burden on health services [2]. In humans, influenza H7N9 virus infection caused a progressive pneumonia leading to acute respiratory distress syndrome (ARDS) reminiscent of highly pathogenic avian influenza (HPAI) H5N1 infection [3]. ARDS is a severe form of acute lung injury resulted from various causes such as increased inflammatory burden [4]. Indeed, there were a great number of studies revealed that influenza H7N9 virus induced a wide variety of proinflammatory cytokines in the lung and serum [5–8]. However, the exact mechanism of the inflammation by H7N9 infection remains unclear.

Necroptosis is a newly discovered pathway of regulated necrosis that requires receptor-interacting protein kinase 3(RIP3) and mixed lineage kinase domain-like protein (MLKL) which is induced by death receptors, interferons, toll-like receptors, intracellular RNA and DNA sensors, and probably other mediators [9–13]. A whole-genome screen for mediators of RIP3-mediated necroptosis identified several regulators of antiviral and antimicrobial innate immunity [14], and numerous studies have shown that RIP3 and the related kinase RIPK1 control necrotic and inflammatory outcome after infection by certain bacteria and viruses, as well as after exposure of cells to select pathogen-associated molecular patterns (PAMPs) and cytokines [15–17].

Here, we report that lack of RIP3 leads to apparently decreased cytokine expression and alleviative susceptibility to influenza A (H7N9) virus-induced acute lung injury in mice. We showed that there was not any defects in antiviral immunity in RIP3 knock out mouse model or any distinct extent in pulmonary tissue destruction following infection. Rather, RIP3-mediated necroptosis-independent inflammation is the key matter during H7N9 virus infection. Collectively, we found that RIP3-mediated activation of caspase-1 rather than necroptosis-dependent inflammation was responsible for aggressive inflammation in influenza A (H7N9) virus-infected mice.

## RESULTS

### RIP3 deficiency attenuates susceptibility in mice infected with Influenza A (H7N9) virus despite viral clearance

**Figure 1:**
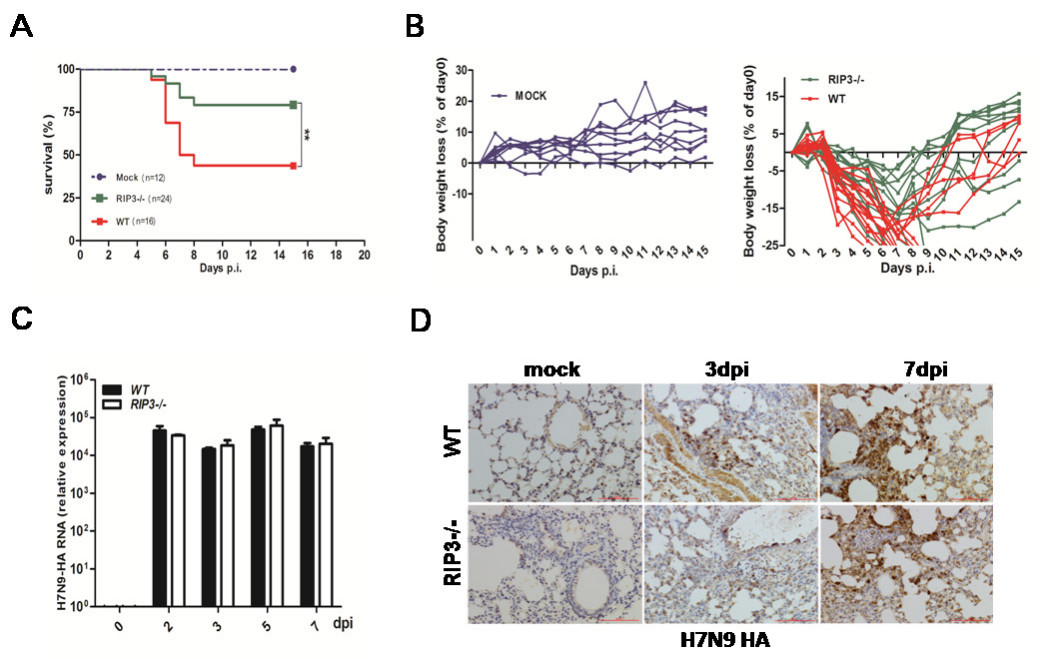
RIP3 deficiency promotes survival in mice infected with H7N9 virus despite virus control **A**. and **B**. (A) Survival and (B) body weight changes of H7N9 virus-infected WT (*n* = 16) and RIP3−/− (*n* = 24) mice compared to PBS-inoculated WT and RIP3−/− mice (*n* = 12) (**p < 0.01). **c**. H7N9 virus HA gene of lung tissues was measured by qPCR at 2, 3, 5 and 7 days p.i. **D**. H7N9 virus HA protein of lung tissues was detected by immunostaining. Scale bar indicates 100μm. Data collected from three independent experiments (*n* = 6 mice per genotype per time point) was shown as mean±SEM. Each line in (B) represents an individual mouse.

To assess possible function of RIP3 to viral infections, influenza H7N9 virus was used to infect wild type (WT) and RIP3−/− mice with a sublethal dose. During the experiment, weight loss, pathologic and virologic parameters of mice were monitored. After intranasal infection with H7N9 virus, more than 75% of RIP3−/− mice survived by day 15 postinfection (p.i.), in contrast, WT mice had decreased survival (*p* < 0.01) and less than 50% mice were recovered (Figure 1A). Both genotypes mice consistently reached the lightest point of their weight at 7-9 days postinfection, notably, at the 7-9 days time frame the weight loss of WT mice was significantly worse (Figure 1B). Furthermore, to investigate whether viral replication responsible for the significant difference in host survival, the mRNA levels of H7N9 virus hemagglutinin (HA) was measured by quantitative RT- PCR. As shown in Figure 1C, H7N9 HA mRNA could be detected from 2 days postinfection, and there was a similar level of HA expression between WT and RIP3−/− mice. In addition, the H7N9 virus-positive cells staining for HA were quantified in the lung tissue sections on day 3 and day7 postinfection and showed no significant difference between genotypes in the virus burden (Figure 1D). These findings indicate that the lethality of H7N9 virus infection in mice is viral control-independent.

### Pulmonary inflammation was alleviated in RIP3−/− mice challenged with H7N9 virus

**Figure 2:**
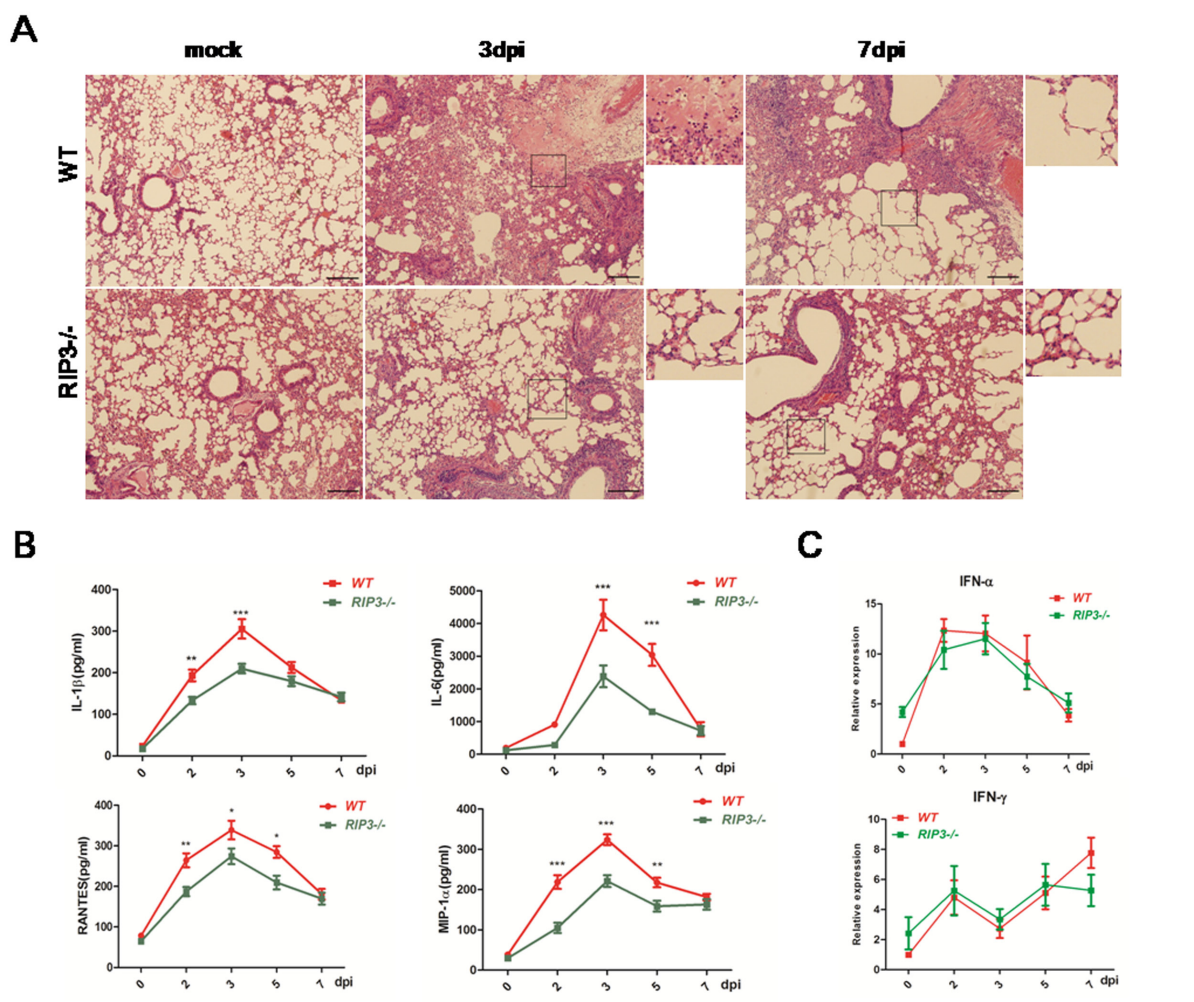
Pulmonary inflammation is alleviative following H7N9 virus infection in RIP3−/− mice **A**. H&E sections through bronchioles showing edema, infiltration with inflammatory cells, and alveolar collapses. Images shown are representative of 6 mice per genotype per time point collected from three independent experiments. Scale bar indicates 100μm. **B**. IL-1β, IL-6, RANTES and MIP-1 secreted in BALF were detected by using ELISA. **C**. IFN-α and IFN-γ expression of lung tissues were measured by qPCR. Data collected from three independent experiments was shown as mean±SEM. (*p < 0.05, **p < 0.01, ***p < 0.001; *n* = 6 mice per genotype per time point).

To determine the effect of RIP3 on histopathological changes of lungs, tissue sections obtained from PBS-treated mice as well as those from H7N9 virus infected WT and RIP3−/− mice on days 3 and 7 p.i. were examined. The lungs of mice infected with or without H7N9 virus showed significantly difference. The H7N9 virus-challenged mice exhibited peribronchiolar inflammation, edema, and viral pneumonia. However, compared with WT mice, RIP3−/− mice had fewer regions of edema, infiltration with inflammatory cells, and alvelolar collapses on days 3 and 7 p.i (Figure 2A). Moreover, the pulmonary expression of cytokines and chemokines were measured. The secretions of IL-1β, IL-6, RANTES and MIP-1 in BALF were significantly decreased in RIP3−/− mice compared with WT mice on days 3 and 7 p.i. (Figure 2B). Consistent with above findings, the mRNA levels of these cytokines and chemokines of lung tissues showed a similar trend for increasing expression following infection in both genotypes mice though the cytokine levels was also lower in RIP3−/− mice than which in WT mice (data not shown). Whereas, the expression of IFN-α and IFN-γ had no difference between genotypes (Figure 2C), which indicated that there was no intrinsic defect in RIG-I signaling in the deficiency of RIP3. Collectively, these results indicate that RIP3 plays a role in promoting inflammatory response in the lung following H7N9 virus infection.

### RIP3 expression is increased in mice exposed to H7N9 virus

**Figure 3:**
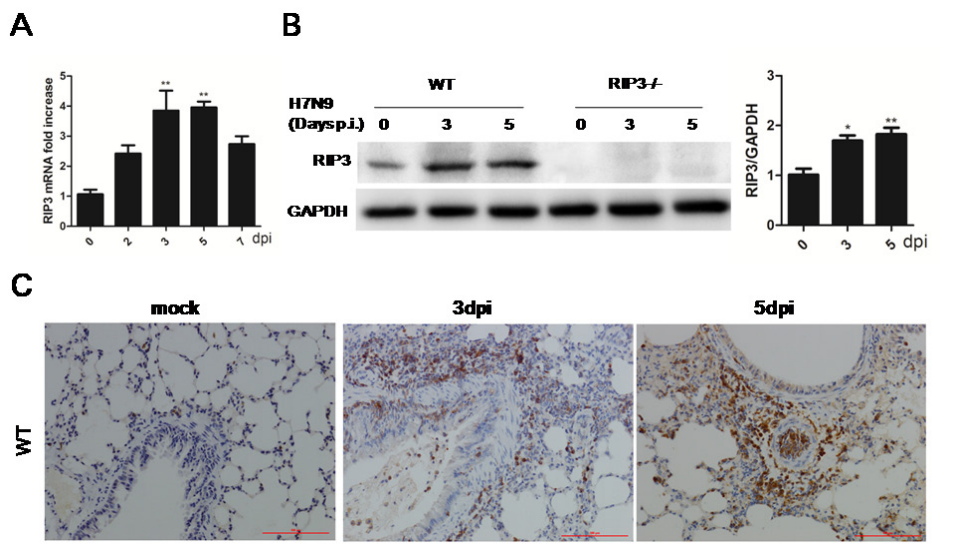
RIP3 is induced in lung tissues during H7N9 virus infection **A**. Relative RIP3 mRNA expression in WT mice following H7N9 virus infection at 2, 3, 5 and 7 days. (**p < 0.01; mean±SEM; *n* = 3-4 mice per time point). **B**. Immunoblot analysis of RIP3 in lung tissue at 3 and 5 days p.i. (representative of three independent experiments). Each lane corresponds to an individual mouse. Lane-loading differences were normalized by levels of GAPDH. (*p < 0.05, **p < 0.01; mean±SEM; *n* = 3 mice per time point). **C**. RIP3 in the lung tissues was detected by immunostaining, which are representative of three to four mice per time point collected from three independent experiments. Scale bar indicates 100μm.

To assess the RIP3 expression pattern, RIP3 mRNA and protein levels were measured, and we found that there was an strongly induced expression in the lung tissues during H7N9 virus infection (Figure 3A and 3B). Besides, immunohistochemical staining for RIP3 showed a similarly elevated expression on days 3 and 5 p.i. (Figure 3C) Notably, the strongest positive signal was mostly seen in infiltrating cells though there was little immunopositive signal in airway epithelial cells (Figure 3C). Since RIP3 was dramatically increased in lung tissues when exposed to H7N9 virus and was primarily produced by inflammatory cells, we reasoned that RIP3 is required for H7N9 virus-induced cytokine release. Additionally, considering that there was also a small number of expression of RIP3 in airway epithelial cells, RIP3 may also had a certain role in epithelial tissues.

### RIP3 loss has a limited effect on maintaining lung tissue integrity in mice following H7N9 virus infection

**Figure 4:**
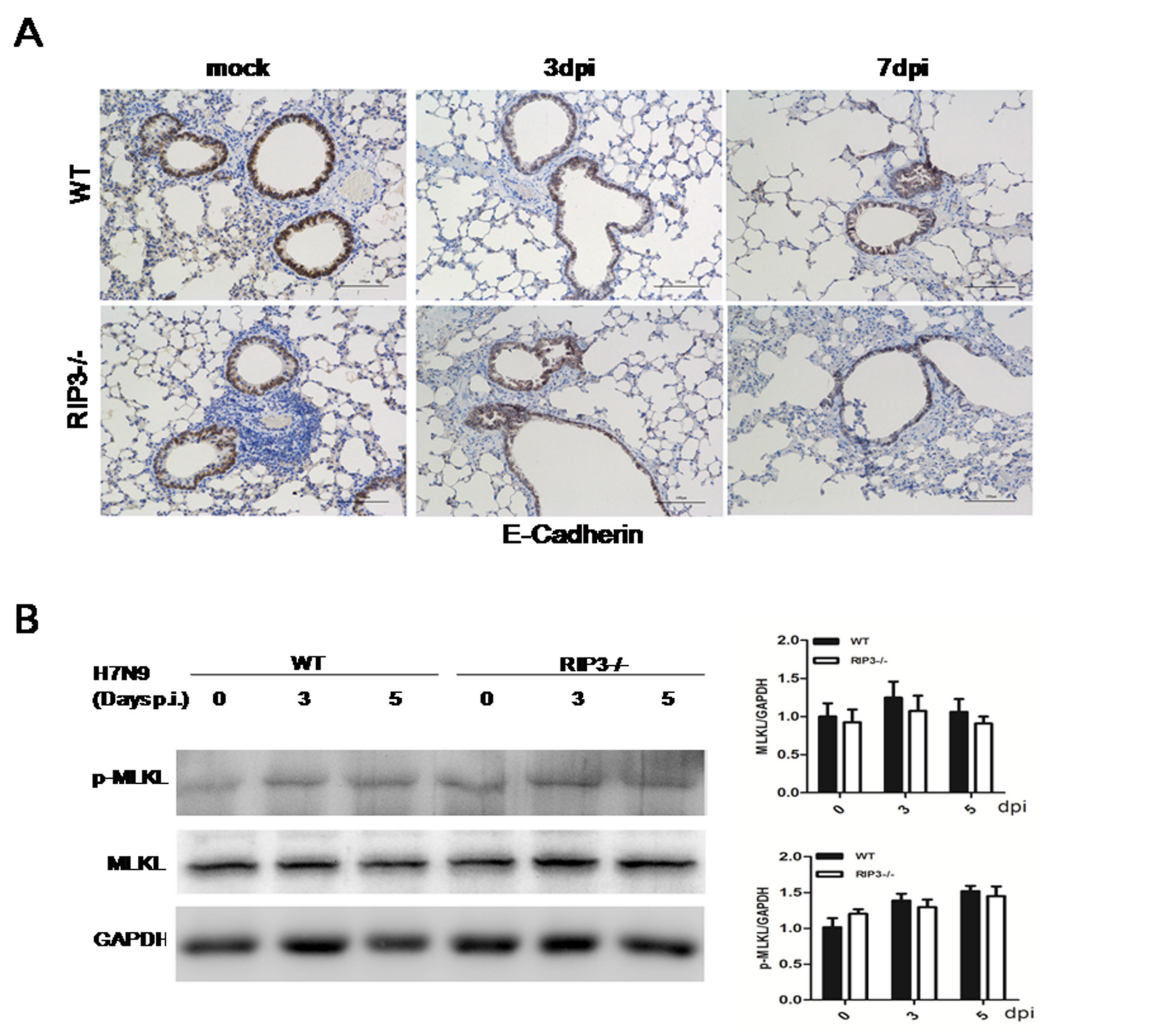
Necroptosis is not the leading cause of distinct lethality in mice challenged with H7N9 virus **A**. Immunohistochemical analysis of lung sections showing bronchioles epithelial cells shedding. Images shown are representative of three to four mice per genotype per time point collected from three independent experiments. Scale bar indicates 100μm. **B**. Immunoblot analysis of MLKL in lung tissue at 3 and 5 days p.i. (representative of three independent experiments). Each lane corresponds to an individual mouse. The data were normalized by levels of GAPDH. (mean±SEM; *n* = 4 mice per genotype per time point).

To determine the protective or destructive role of RIP3 in airway epithelial tissues, immunohistochemical analysis was performed in the lung sections of PBS-treated and virus-challenged mice on days 3 and 7 p.i. IHC staining for the E-Cadherin, which was found in epithelial tissue, playing an important role in cell adhesion and forming adherens junctions to bind cells within tissues together, was probed. Compared with PBS-treated groups, WT or RIP3−/− mice chanlleging H7N9 virus exhibited obvious bronchiolar epithelial damage with extensive epithelial shedding (data not shown). Nevertheless, the extent of epithelial tissues injury showed no significant difference between genotypes (Figure 4A). Bronchioles of both genotypes mice prior to infection had the similar, normal epithelial cell lining (Figure 4A). Since immunohistochemical analysis of lung sections uncovered characteristics of necrotizing bronchitis, we further investigated the effect of RIP3 on necrosis-dependent pathway of inflammation following H7N9 virus infection. Thus, RT- qPCR and immunoblot analysis of MLKL were performed. The mRNA levels of MLKL in the lung tissues had no significant difference between WT and RIP3−/− groups after H7N9 virus infection (data not shown), correspondingly, no statistically significant changes in MLKL protein levels were detected in the lung tissue lysate either (Figure 4B). RIP3-dependent necroptosis is widely believed to exacerbate disease, in this study, however, we found few evidences to support this view. We had shown that RIP3 deficiency had a restricted role in maintaining tissue homeostasis, and the decreased mortality to H7N9 virus in RIP3−/− mice was unlikely to be due to the absence of RIP3-dependent necrosis, as both RIP3−/− and WT mice exhibited similar destructive changes to their airway architecture and the RIP3-MLKL-mediated necroptosis pathway was not burst as expected, which suggested that RIP3 possibly had a more complicated role in promoting inflammation other than necroptosis-dependent pathway.

### RIP3-mediated activation of caspase-1 is responsible for cytokine expression

**Figure 5:**
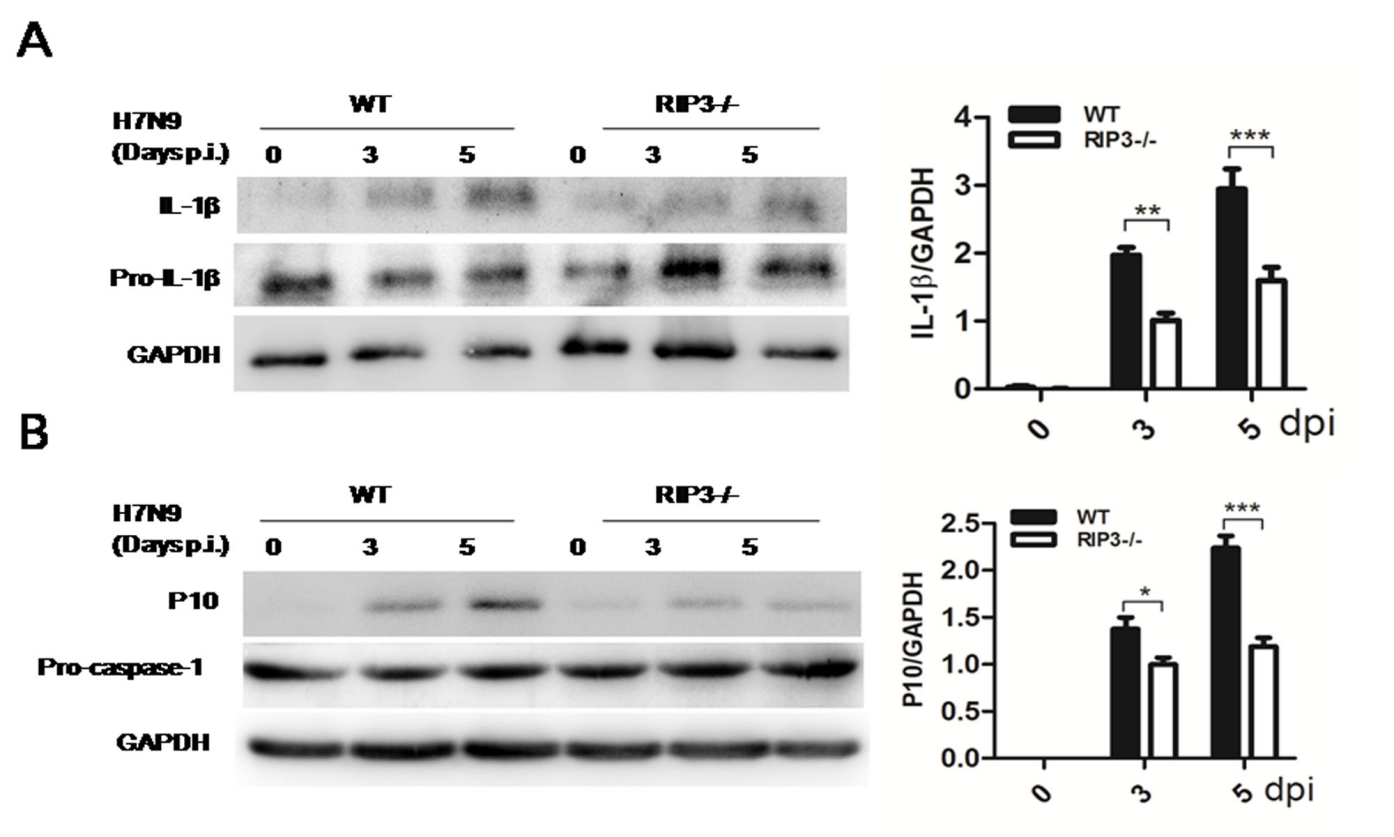
RIP3 promotes the activation of caspase-1 **A**. Immunoblot analysis of IL-1β and pro-IL-1β in lung tissue at 3 and 5 days p.i. (representative of three independent experiments). **B**. Immunoblot analysis of p10 and pro-caspase-1 in lung tissue at 3 and 5 days p.i. (representative of three independent experiments). The data were normalized by levels of GAPDH. (*p < 0.05, **p < 0.01, ***p < 0.001; mean±SEM; *n* = 4 mice per genotype per time point).

To investigate the possible other mechanism of RIP3-dependent cytokine expression, IL-1β, an important pro-inflammatory mediator, was measured again. IL-1β secretion was apparently lower in RIP3−/− mice (Figure 2B), however, considering that ELISA may not reliably distinguish mature IL-1β from pro-IL-1β, we used immunoblotting to better assess levels of mature IL-1β. Consistently, immunoblot analysis confirmed that mature IL-1β protein amounts were markedly increased in WT mice on days 3, 5, p.i. compared with RIP3−/− groups (Figure 5A). Notably, mature IL-1β production requires caspase-1 activation which can be measured by cleave p10 fragment. Then, we detected p10 fragment protein using immunoblotting and dramatically reduced amounts of which were observed in RIP3−/− mice after H7N9 virus infection (Figure 5B). These data indicated that RIP3 can promote inflammatory cytokine expression via the activation of caspase-1, moreover, RIP3 has been shown to exerts key functions in the activation of caspase-1.

## DISCUSSION

Necroptosis, a proinflammatory form of cell death, is critically regulated by the serine-threonine kinase RIP3, one of the members of receptor interacting protein kinase family [18, 19]. RIP3 interacts with a closely related upstream kinase RIP1 to form a necrosis-inducing protein complex termed the necrosome which is essential for recruitment and activation of the downstream RIP3 substrate mixed lineage kinase domain-like (MLKL) [20]. Whereafter, MLKL forms oligomers and translocate to plasma membrane and eventually induce membrane disrupture [21, 22]. Necrotic membrane rupture causes the leakage of cellular contents including danger-associated molecular patterns (DAMPs) which is believed to be the key driver of inflammatory diseases.

The contribution of necroptosis to inflammation in human diseases or in many widely used mouse models of disease is less clear. To date, investigators have largely inferred that necroptosis exacerbates tissue injury because the inhibition of RIP3 has been shown to be beneficial in many tissue injury models, including pancreatitis [23, 24] atherosclerosis [25], retinal degeneration [26], kidney ischemia-reperfusion injury [27], Gaucher's disease [28], myocardial infarction[29, 30], and systemic inflammatory response (SIRS) syndrome [31]. Therefore, inhibition of necroptosis has been suggested as a novel therapeutic approach for reducing inflammation. However, emerging data suggest that RIP3 can also facilitate inflammation in a necroptosis independent manner [32–34].

In humans, both necrosis and apoptosis of distal pulmonary epithelia were described as a classical feature of H5N1 or H1N1 influenza-induced acute respiratory distress syndrome [35, 36]. Moreover, Rodrigue-Gervais IG reported that genetic deletion of RIP3 rescued cIAP2-deficient mice from influenza-induced lethality [37]. A more recent study demonstrated that IAV activates parallel pathways of MLKL-driven necroptosis and FADD-mediated apoptosis, with the former reliant on RIP3 kinase activity and neither on RIPK1 activity [38, 39]. They also found that mice deficient in RIP3 or doubly deficient in MLKL and FADD, but not MLKL alone, are more susceptible to IAV than their wild-type counterparts, revealing an important role for RIP3-mediated apoptosis in antiviral immunity. Collectively, these results outline RIP3-activated cytolytic mechanisms essential for controlling respiratory IAV infection [38]. In the present study, it was found that RIP3 deficiency can protect mice from the infection of by influenza H7N9 virus, which was consistent with the previous reports [35–39]. However, the mechnism of RIP3 effect was focused on the caspase 1/IL1β signaling, which sugguested the RIP3 invovled necrosis independent manner. In fact, there are some evidences of RIP3 activation of caspase-1/IL-1β signaling. RIP3 has been implicated to accelerate pro-IL-1β processing in dendritic cells (DCs) and macrophages. Production of the innate inflammatory cytokine IL-1β requires de novo synthesis of pro-IL-1β via a NF-κB-dependent manner as well as the cleavage and maturation of IL-1β by caspase-1. As reported that enhanced NLRP3 inflammasome activation and increased mature IL-1β secretion was observed in LPS-primed cIAP1−/− cIAP2−/− and XIAP−/− macrophages or DCs [40]. Similar results were also obtained in caspase-8 deficiency DCs. Mice with DC-specific caspase-8−/− led to greatly elevated expression of IL-1β and increased activation of NLRP3/ASC/caspase-1 inflammasome which could be reversed by ablation of RIP3 [41]. Strikingly, in these settings the maturation of pro-IL-β and activation of inflammasome is through a RIP3-dependent mechanism rather than necrosis. However, there is discrepant interpretation on whether accelerated inflammatory reaction was due to enhanced RIP3-dependent necroptosis or via distinct mechanisms.

The RIP3 field is filled with controversy. Besides influenza virus, the roles of RIP3 in other diseases were investigated [42–44]. Most recently, Newton K, et al. reported that the loss of RIP3 had no effect on lipopolysaccharide-induced sepsis, cerulein-induced pancreatitis, hypoxia-induced cerebral edema, or the major cerebral artery occlusion stroke model [45]. However, kidney ischemia-reperfusion injury, myocardial infarction, and systemic inflammation associated with A20 deficiency or high-dose tumor necrosis factor (TNF) were ameliorated by RIPK3 deficiency [45]. Thus, it is believed that RIP3 might play diverse roles in different disease progression.

In the conclusion, our results indicated that RIP3 deficiency protected that mice from by influenza H7N9 virus infection by downregulating caspase 1/IL1β signaling, which provided edivence of the RIP3 invovled necroptosis independent manner. The the discrepancy might be resulted from the subtype of influenza virus or other interfering factors in virus infection protocol, and further study is needed.

## MATERIALS AND METHODS

### Mice

RIP3−/− mice kindly provided by Prof. Wang XD [24] were maintained on a 12h light/dark cycle at 22°C, given water adlibitum and fed standard laboratory chow. Seventy two RIP3−/− mice and 72 weight matched wild type littermate mice were used for experiments at 7-9 wk of age (bodyweight: 18 to 22g). For both RIP3−/− and WT mice, mice were equally divided to AAV infection group and control group randomly, and every 6 mice for 0, 1, 2, 3, 5, 7dpi group respectively. All experiments were performed in the animal Biosafety Level 3 Laboratory (BSL-3), and the procedures were conducted with the approval by the Animal Research Committee of Second Military Medical University.

### Virus infection and lung tissue sampling

Influenza virus (A/Fujian/S03/2015(H7N9), Gene bank accession KY286424-KY286431) was obtained from the Center for Disease Control (CDC) of Shanghai and propagated in embryonated chicken eggs for 48 hours at 35°C and the virus was stored at −80°C before use. The study with the H7N9 influenza virus was carried out in the BSL-3 containment facility.

Animals were matched according to their age and sex, and were anesthetized by inhalation with isoflurane before infected intranasally with a sublethal (0.5LD50) dose of H7N9 in 40μl PBS. At the specific time-point, animals were euthanized by isoflurane, and then we exposed the trachea and cannulated it. Following these procedures, the bronchoalveolar lavage fluid (BALF) was collected by injecting and pooling 0.6ml cold, sterile PBS twice. As well, mice in the same group were euthanized and the whole lungs were obtained. Among these, the right lung lobes were used for histopathological analysis and the left ones were used for total RNA isolation and immunoblotting.

### Histopathology and immunohistochemistry

The lung lobes of the animal in each experimental group were collected on days 0, 2, 3, 5, and 7 postinfection (p.i.), and were perfused with 10% neutral-buffered formalin (NBF) at 4°C for 48 hours. Then the fixed tissues were immersed in 30% sucrose in 0.1M PBS at 4°C overnight. Finally the tissues were embedded in paraffin and sections with 4μm thickness were obtained and staining with hematoxylin and eosin (H&E) or with immunohistochemical (IHC) staining for H7N9 virus by using hemagglutinin (HA, Sino Biological Inc., BeiJing, China), RIP3(Cell Signaling Technology Inc., MA, USA), MLKL and E-Cadherin (ABclonal, WuHan, China) at 4°C overnight, followed by incubation with HRP-conjugated goat anti-rabbit antibody (1:1000 dilution, KPL) at room temperature for 60 min. Finally, 3, 3′-diaminobenzidine tetrahydrochloride (DAB) was used for signal development, and 20% hematoxylin was used for counterstaining.

To calculate the numbers of immunopositive signals on the antibodies stained sections, five microscopic fields in every section around the bronchial tube were photographed randomly, and then manually outline the area of positive signals per fields. The percentage of the area with positive staining coverage was calculated via the Aperio ImageScope software.

### Lung cytokine and chemokine analysis

The concentrations of cytokines and chemokines in BALF including interleukin-1β (IL-1β), interleukin-6 (IL-6), regulated upon activation, normal T cell expressed and secreted (RANTES), and macrophage induced protein-1 (MIP-1) were measured by using an ELISA kits (BMS6002, BMS603/2, BMS6009INST, 88-56013; eBioscience Inc., CA, USA), whose procedures were according to the manufacturer's protocols. The samples plates were read on the BioTek instruments using the Gen5 Take3 Module software. The absorbance at 450 nm was measured and the data were analyzed based on a standard curve.

### Quantitative real-time PCR

The mRNA levels of the H7N9 virus HA and some cytokines or chemokines were measured on days 0, 2, 3, 5, and 7p.i. Lung tissues were homogenized in the Trizol Reagent (Invitrogen, CA, USA) in order to extract the total RNA according to the manufacturer's instructions. Complementary DNA (cDNA) was reverse transcribed with the PrimeScript™ RT Master Mix (Takara Bio, Shiga, Japan). The reverse transcription product of all targeted genes was incubated with the SYBR^®^ Premix Ex Taq™ kit (Tli RNaseH Plus) (Takara Bio), together with the specific primer, and the mixture system was placed in a 96-well optical plate and was run on a Step One Plus real-time PCR machine (AppliedBiosystems, CA, USA). The parameters of the thermal cycling were 95°C for 7min, followed by 40 cycles at 95°C for 15s and 60°C for 1 min. GAPDH was considered as the reference gene to normalize all the genes expression. All the primer sequences can be seen in Supplementary Table 1.

### Immunoblotting

Lung tissue samples were frozen in −80°C condition before use. 0.5g of the tissue was obtained and homogenized in the RIPA lysis buffer in the presence of proteinase inhibitor cocktail (Complete mini, Roche). The concentration of the total protein was measured by BCA protein assay kit (Bio-Rad Laboratory, CA, USA). 20 μg protein of the tissue were loaded onto a 10% sodium dodecyl sulfate polyacrylamide gel (SDS-PAGE) and separation by electrophoresis. After proteins were transferred onto a polyvinylidene difluoride (PVDF) membranes (ImmobilonP-SQ, Millipore, MA, USA), the PVDF membranes were blocked with 5% non-fat milk in Tris-buffered saline containing 0.05% Tween 20 (TBS-T) for 2 hours at room temperature. Then the membranes were incubated with the specific primary antibody at 4°C overnight, the membranes were washed 3 times with TBS-T, followed by incubation with secondary horseradish peroxidase (HRP)-conjugated antibody (1:5000 dilution, KPL Inc., MD, USA) for 2 hours at room temperature. After washing 3 times, the protein bands were visualized by chemiluminescence with a phototope-HRP detection kit (Pierce, Rockford, IL, USA) through the Gene Gnome HRImage Capture System (Syngene, Frederick, MD, USA) and the density of band was analyzed by the Image J software. The primary antibodies used in the study were: RIP3, IL-1β, pro-IL-1β (Cell Signaling Technology), and P10, pro-caspase-1 (Abcam, MA, USA) and MLKL, GAPDH antibody (ABclonal). All the data were normalized to GAPDH.

### Statistical analysis

Data analysis was performed via GraphPad Prism software version 5.0 and represented as mean ± standard deviation (SD) or SEM. For comparisons between groups, one-way analysis of variance (ANOVA) combined with a two-tailed t test, Mann-Whittney U test or chi-square test were performed, and log rank test was used to analyzed the survival curves. *P* < 0.05 was defined statistically significant.

## SUPPLEMENTARY MATERIALS TABLE


