# Supplementary material for: RIP3 deficiency ameliorates inflammatory response in mice infected with influenza H7N9 virus infection
Source: Oncotarget. 2017 Mar 8;8(17):27715–24. doi: 10.18632/oncotarget.16016 (PMC5438603; doi:10.18632/oncotarget.16016)
Supplement: Supplementary file 1 [file oncotarget-08-27715-s001.pdf]

RIP3 deficiency ameliorates inflammatory response in mice infected with influenza H7N9 virus infection

Supplementary Material

Supplementary Table 1: The sequences of primers for real-time qPCR.

Primers	Sequences
H7N9-HA	Forward: 5′-AGAAATGAAATGGCTCCTGTCAA-3′
	Reverse: 5′-GGTTTTTTCTTGTATTTTATATGACTTAG-3′
RIP3	Forward: 5′-ACACGGCACTCCTTGGTATC-3′
	Reverse: 5′-CCGAACTGTGCTTGGTCATA-3′
MLKL	Forward: 5′-CCCGAGTTGTTGCAGGAGAT-3′
	Reverse: 5′-TCTCCAAGATTCCATCCGCAG-3′
IFN-α	Forward: 5′- TCTGATGCAGCAGGTGGG-3′
	Reverse: 5′- AGGGCTCTCCAGACTTCTGCTCTG-3′
IFN-γ	Forward: 5′- AAGTTTGAGGTCAACAACCCAC -3′
	Reverse: 5′- GCTGGCAGAATTATTCTTATTGGG -3′
TNF-α	Forward: 5′- GTCCCCAAAGGGATGAGAAGTT -3′
	Reverse: 5′- GTTTGCTACGACGTGGGCTACA -3′
IL-1β	Forward: 5′- TGTGAAATGCCACCTTTTGA -3′
	Reverse: 5′- GGTCAAAGGTTTGGAAGCAG -3′
IL-6	Forward: 5′- TCTTGGGACTGATGCTGGTG -3′
	Reverse: 5′- TGCCATTGCACAACTCTTTTCT -3′
MCP-1	Forward: 5′- CTCAGCCAGATGCAGTTAACG -3′
	Reverse: 5′- GGGTCAACTTCACATTCAAAGG -3′
GAPDH	Forward: 5′- CAATGAATAGGGCTACAGCA -3′
	Reverse: 5′- AGGGAGATGCTCAGTGTTGG -3′
